# Cytoplasmic Ribonucleoprotein Foci in Eukaryotes: Hotspots of Bio(chemical)Diversity

**DOI:** 10.1155/2012/504292

**Published:** 2012-05-27

**Authors:** Carla Layana, Paola Ferrero, Rolando Rivera-Pomar

**Affiliations:** ^1^Centro Regional de Estudios Genómicos, Universidad Nacional de La Plata, CP 1888 Florencio Varela, Argentina; ^2^Departamento de Ciencias Básicas y Experimentales, Universidad Nacional del Noroeste de Buenos Aires, Avenida Calchaquí 5900, CP 1888 Buenos Aires, Pergamino, Argentina

## Abstract

The life of an mRNA from transcription to degradation offers multiple control check points that regulate gene expression. Transcription, splicing, and translation have been widely studied for many years; however, in recent years, new layers of posttranscriptional and posttranslational control have been uncovered. They involve the regulation of the metabolism of mRNA in cytoplasmic foci. They are collections of ribonucleoprotein complexes that, in most cases, remain still uncharacterized, except the processing bodies (PBs) and stress granules (SGs), which have been studied (and reviewed) in detail. A challenging prospective is to know how many different classes of foci exist, which functions they support, how are they formed, and how do they relate one to each other. Here, we present an update of the component of the different granules, a possible function, and hypothesis on their *in vivo* dynamics related to translational control.

## 1. Introduction

In recent years, several cytoplasmic foci/granules that contain proteins and RNA have been described. Two of them have been studied in more detail as they are related to mRNA silencing: stress granules (SG) and processing bodies (PB). SG are repressed mRNPs transiently induced in response to cellular stress. They range from 0,5 to 5 *μ*m [1]. PB are discrete RNP cytoplasmic foci of 0,1-2 *μ*m where the machinery of RNA interference, degradation and storage locates. In PB the mRNAs are forming mRNP complexes either repressing translation, in degradation complexes or stored for further use [2, 3]. SG and PB have been shown to share a growing number of proteins that are added in a day-to-day basis to the list of their components. SG, PB and other cytoplasmic foci are highly dynamic structures, although PB are quite stable over the time [4]; see also Supplementary Movie 1 available online at doi:10.1155/2012/504292. They are in a dynamic steady state with other mRNPs, such as polysomes in response to the translational state of the cell [5]. Although we do not intend to extensively review SG and PB, which have been matter of fine reviews in the last years [6–10], we will overview their functions before we address neglected issues and hypothesis.

## 2. Stress Granules

Translation initiation is the key regulatory step of translational control. Therefore, it is the most sensitive step to changes in the cellular environment, including stress. A key step in translation initiation inhibition is the phosphorylation of eIF2*α*, which results in an increase on the affinity of eIF2-GDP for eIF2B, sequestering this factor to prevent new round of translational initiation [11]. During this process, translation is inhibited and polysomes become released from the mRNA leading to the accumulation of inactive mRNPs in SG. The SG are in equilibrium with active polysomes. Protein elongation inhibitors, such as cycloheximide, prevent the assembly of SG by blocking the polysomes in an inactive state, while protein initiation inhibitors promote the formation of SG [8].  Table 1 shows the components of SG described up to now. They can be classified in three main groups as follows.

(1) Core components: stalled initiation complexes (polyadenylated mRNAs and translation factors eIF4E, eIF4A, eIF4G, eIF3, eIF2, PABP, and proteins of the small ribosome subunit).

(2) RNA-binding proteins associated to silencing and transcript stability: TIA-1, TIAR [12], FAST, Argonaute [13], CPEB, smaug, DExD/H-box RCK/p54 (o Dhh1), XRN1 [5].

(3) RNA-binding proteins associated to mRNA metabolism either translation of degradation such as G3BP [14] and Staufen [15].

The key concept regarding SG is that they are responsible of protecting the mRNA during cell stress, altering the composition of the mRNPs in a reversible manner. As soon as the cell recovers, the mRNPs regain their translational capacity.

## 3. Processing Bodies

These structures have been described many times since 1997, when Bashkirov et al. observed that the exonuclease Xrn1 is located in small granular structures in the cytoplasm of mammalian cells and call them “Xrn1 foci” [17]. Later on, the decapping enzyme Dcp2 was also described to occur in cytoplasmic foci [18]. Contemporary, Eystathioy et al. have described that a protein associated to neuropathy named GW182 occurs in cytoplasmic speckles called GW bodies [19, 20]. Other RNA-related protein, the eIF4E-transporter, was also localized in discrete cytoplasmic foci [16, 21, 22]. Short after, a seminal work of Sheth and Parker established the functional bases of the now called PB that resulted in the same structures described many times before [23]. They demonstrated that PB contains enzymes involved in the degradation of the mRNA. Later one further studies showed that they are also related to miRNA metabolism and can store mRNAs to bring them back to polysomes (reviewed in [3, 24]. They include, different than SG, neither ribosomal proteins nor translation factors, except eIF4E. They do not present either the exosome components [25]. eIF4G and PABP were found in yeast PB, although at low level and in stress conditions resulting on glucose deprivation [26]. Proteins and mRNA can reversely go in and out of PB [25]. The relationship of PB and polysomes is demonstrated by the blocking of PB formation by cycloheximide. A summary of the components in different organisms is shown in Table 2. The occurrence of such large and diverse set of proteins (and the list continuously grows up) suggests that PBs are involved in a plethora of posttranslational processes regulating gene expression, such as mRNA degradation and silencing. mRNA degradation starts with the shortening of the poly-A tail—the deadenylation. In eukaryotes, there are several complexes involved in the process: PARN2-PARN3 initiates the process, which continues with the action of the CAF1-CCR4-NOT complex. Later on, mRNA degradation continues by nucleolytic cleavage on both ends. 3′ → 5′ degradation is catalyzed by the exosome and the SKI complex, while 5′ → 3′ degradation requires previous decapping by DCP2 and the coactivator DCP1 and the action of the exonuclease XRN1. All these enzyme localize in PB. There are several evidences indicating that mRNA degradation occurs in PB.

(i) The assembly of PB depends on mRNA, as RNase treatment of the cells induces the disappearing of PB [27, 28].

(ii) Inhibition or removal of the deadenylase Ccr4 reduces the number and size of PB, while the removal of the downstream-acting enzymes Xrn1 and Dcp1 does not affect the stability of PB [21].

(iii) mRNA degradation intermediates are present in PB [23].

Therefore, one can conclude that mRNA degradation occurs in PB and depends on the existence of degradation enzymes and mRNA degradation intermediates [21, 23, 25, 29]. Many of the PB components are not restricted to the foci and also are present in the soluble cytoplasm and nuclei, suggesting that the different processes might start before the mRNAs entry into PB. PBs are also related to mRNA quality control mechanisms, such as nonsense-mediated decay (NMD). The detection of premature termination in the cells by spotting an mRNA with an abnormal stop codon is mediated by a surveillance complex composed by UPF1, UPF2 y UPF3, additional proteins, namely, SMG1, and SMG5-7 [30–32]. As soon as the surveillance complex is assembled, the degradation enzymes (Dcp1, Xrn1) are recruited to the mRNA in PB. Although the degrading enzymes are located in PB, the mechanism of recruitment is unknown. In silencing, there are two types of small mRNAs that regulate posttranscriptional gene expression: siRNAs and miRNAs. Despite the different mechanism of silencing, in both cases participate the protein Argonaute (Ago) and the RISC (RNA-induced silencing complex). In the case of siRNAs, Ago produces an endonucleolytic cleavage of the mRNA to promote degradation by the 3′ → 5′ and 5′ → 3′ decay machinery in PB. In the case of the miRNAs, they recruit Ago to direct the repressed mRNA to degradation mediated by the PB proteins GW182, CCR4-CAF1-NOT1, DCP2, DCP1, and XRN1.

## 4. The Cycle of an mRNA in the Cell: SG, PB, Polysomes, and the Unknown Intermediates

**Figure 1:**
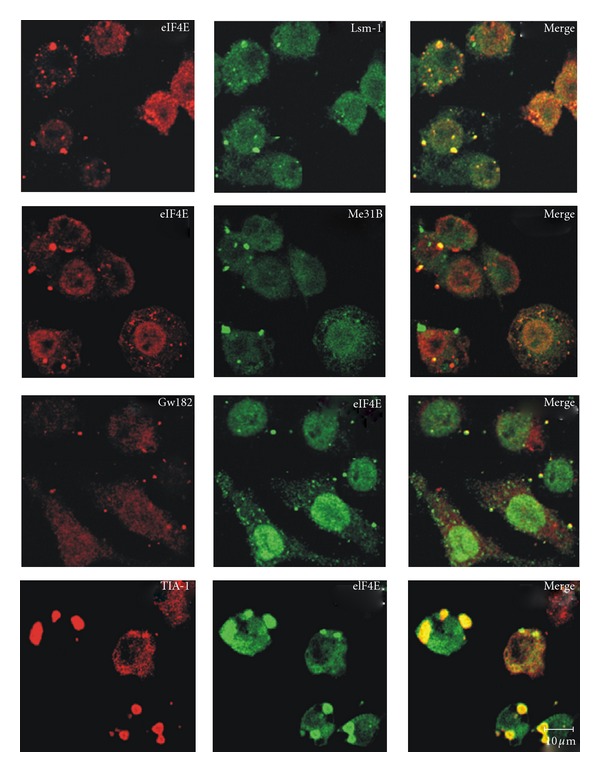
Colocalization of eIF4E with components of PBs and SGs shows a diversity of cytoplasmic foci quality. The experiment shows that, in every case, the granules contain both components or either one or the other in different quantities. This would represent intermediates or different forms of eIF4E-containing foci. *Drosophila melanogaster* S2 cells were transfected with proteins fusion CFP-Lsm-1 or CFP-Me31B. GW182, eIF4E, and Rox8 (the TIA-1 ortholog in *Drosophila*) were revealed using antibodies against GW182, anti-V5, and anti-TIA-1, respectively. In the bottom panel (row 4), the cells were prestressed with arsenite for 30 minutes.

**Figure 2:**
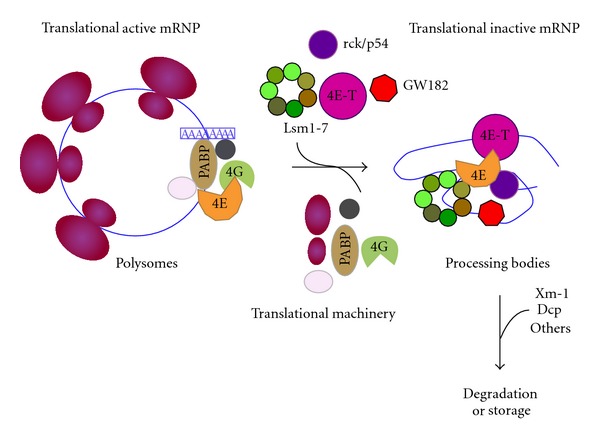
Relationship among active polysomes and PBs. The recruitment of active polysomes to PB implies the removal from the mRNA of the translation factors by translational repressors. Some of them have been demonstrated to interact with eIF4E *in vivo* (rck/p54 and eIF4E-T, [16]). They further interact and/or recruit the enhancers of decapping Lsm1-7 or the miRNA-related protein GW182 to form the PB. Later on, they assemble the decapping and degradation enzymes and/or the proteins required for silencing and storage into PB. All the intermediate steps of this process can represent different populations of granules coexisting in the cell and visible with different morphology that might reflect a variety of components and/or diverse stoichiometry.

From the previous analysis, one can establish many unsolved aspects on cytoplasmic foci function. One of them is the dynamic of the mRNP remodeling. The current model suggests an active movement of mRNPs from and to polysomes and from and to SG and PB [33]. However, how does it happen and the factors involved are not known. Translationally active mRNAs can interact, in response to errors in translational initiation or to specific recruitment of regulatory proteins, with translational repressors such as Dhh1, Pat 1, Lsm1-7, eIF4E-T. Those factors would promote the replacement of the translational machinery from the mRNA, promote the cap removal and determine degradation [33] or the accumulation of silenced mRNA in PB. Within PB, mRNPs could undergo further remodeling and define a path to follow, including their return to polysomes. In addition, PBs have been shown to interact and exchange components or their own nature with SG (reviewed in [6, 7]) in a process that may result in mRNPs intermediates of unknown nature. Evidence for the diversity of cytoplasmic foci and their components results from immunocytochemistry and colocalization studies. A common factor present in most cytoplasmic mRNPs is the cap-binding protein eIF4E. eIF4E occurs in active polysomes as a translation initiation factor, in SG as part of the stalled initiation complex, and in PB as the only translation factor present there in multicellular eukaryotes. We observed in *Drosophila *S2 cells that eIF4E colocalizes with different pairs of markers, either for PB (GW182, Lsm1, Me31B—an ortholog of the helicase rck/p54) or SG (TIA-1) and that the colocalization does not occur in all foci in the same way (PVF, CL, and RRP, unpublished data and Figure 1). In some cases, the foci contain one, the other, or both components. In the foci that show colocalization of both factors, the relative amount of each component may vary from foci to foci, as judged by confocal microscopy quantification of the colocalized factors (PVF, CL, RRP, unpublished observation). This implies that there are a diversity of granules. An appealing hypothesis is that eIF4E is a common link among different mRNPs, playing different roles depending on their interactors. One plausible function could be that the accumulation of mRNPs in eIF4E-containing foci is a way to regulate the rate of translation in different physiological states (cell cycle phases, developmental stages, circadian rhythms). Moreover, it has been reported that, in mammalian cells, eIF4E interacts in PB with at least two factors, rck/p54 and eIF4E-T [21]. These are simultaneous interactions within the PB and imply that both proteins could contact different domains of the same eIF4E molecule or that they would represent different populations of mRNPs or different functions within the same PB. In either cases, the complexity of the interactions *in vivo *is more diverse than it has been expected. A model for the remodeling of active mRNPs to silence and degradation based on Andrei et al. [21] is depicted in Figure 2. This might require several intermediate states that can be the maturation steps of a mRNA in the way of a PB or within a PB. This would correlate with the large diversity of components and interactions within a cytoplasmic foci and the diversity of the foci within a cell. The understanding of the dynamics of mRNP is far from clear and unpredictable paths remain to be discovered. They will need further research and more sophisticated methods for *in vivo* studies.

## Supplementary Material

Colocalization of eIF4E with components of PBs and SGs shows a diversity of cytoplasmic foci quality. The experiment shows that, in every case, the granules contain both components or either one or the other in different quantities. This would represent intermediates or different forms of eIF4E-containing foci. *Drosophila melanogaster * S2 cells were transfected with proteins fusion CFP-Lsm-1 or CFP-Me31B. GW182, eIF4E, and Rox8 (the TIA-1 ortholog in Drosophila) were revealed using antibodies against GW182, anti-V5, and anti-TIA-1, respectively. In the bottom panel (row 4), the cells were prestressed with arsenite for 30 minutes.
Relationship among active polysomes and PBs. The recruitment of active polysomes to PB implies the removal from the mRNA of the translation factors by translational repressors. Some of them have been demonstrated to interact with eIF4E in vivo (rck/p54 and eIF4E-T, [20]). They further interact and/or recruit the enhancers of decapping Lsm1-7 or the miRNA-related protein GW182 to form the PB. Later on, they assemble the decapping and degradation enzymes and/or the proteins required for silencing and storage into PB. All the intermediate steps of this process can represent different populations of granules coexisting in the cell and visible with different morphology that might reflect a variety of components and/or diverse stoichiometry.

## Figures and Tables

**Table 1:** Components of stress granules.

Protein	Function	Interacting proteins
Ago2	Cleaves interfered RNA	RISC, FXR1
APOBEC3G	Antiviral response	?
Ataxin-2	Translation	PABP-1
Caprin-1	Cell growth	G3BP
CPEB	mRNA repression	RCK, eIF4E, FXR1
DIS1	Unknown	eIF3h
eIF3	Translation	40S, eIF4G
eIF4E	Translation	CPEB, Smaug, eIF4G, 4ET
eIF4G	Translation	eIF4E, eIF3, PABP-1
FAST	Translation	TIA-1
FMRP, FXR1	Translation	Ago2, RISC
FBP, KSRP	mRNA degradation	TIA-1
FUS/TLS	Transcriptional control	Transcriptional machinery
G3BP	Ras signalling	Caprin
HuR	mRNA stabilization	?
IP5K	Signalling	?
Lin28	Developmental control	?
LINE 1 ORF1p	Transposon	?
MLN51	Splicing	Exon junction
PABP-1	Translation	eIF4G, eIF3, ataxina-2
RCK(p54)	mRNA degradation	GE-1, TTP
Plakophilin	Adhesion	G3BP, FXR1
PMR1	mRNA degradation	TIA-1
Pumilio 2	mRNA silencing	?
Rap 55	mRNA silencing	?
Rpb4	Transcription	?
SRC3	Transcription	TIA-1
Staufen	mRNA silencing	?
SMN	RNP assembly	Complejo SMN
TDP-43	Transcription and splicing regulator	eIF4G, eIF3, eIF2, ribosomal proteins, STAU-1, Xnr
TIA-1(rox-8), TIAR	mRNA silencing	FAST, SRC3, PMR1, FBP
TRAF2	Signalling	eIF4G
TTP, BRF-1	mRNA silencing	RCK (p54)
YB-1	Cold shock	?
ZBP1	Localization	?

**Table 2:** Components of processing bodies.

Protein	Function	Organisms
XRN1, Sc Kem1	5′ → 3′ exonuclease	Human, mice, Sc
GW182, Ce AIN-1	miARN function	Human, Dm, Ce
DCP2, Ce DCAP2	Decapping	Human, Dm, Ce, Sc
DCP1, Ce DCAP1	Decapping	Human, Dm, Ce, Sc
Hedls, Ge-1	Decapping coactivator	Human, Dm
Dm CG5208, Pat1	Decapping coactivator	Dm, Sc
EDC3 (Lsm16)	Decapping coactivator	Human, Dm, Sc
Lsm1-7	Decapping coactivator	Human, Sc
RAP55	Putative decapping coactivator	Human
RCK/p54, Dm Me31B, Ce CGH-1, Sc Dhh1	Decapping coactivator, translational control	Human, Dm, Ce, Sc
eIF4E	Translation initiation	Human, rat, mouse, Dm, Sc
eIF4E-T	Translational repression	Human
SMG7	Nonsense mediated decay	Human
SNG5	Nonsense mediated decay	Human
UPF1, Sc Nam7	Nonsense mediated decay	Human, Sc
UPF2	Nonsense mediated decay	Human
UPF3	Nonsense mediated decay	Human
Argonaute	siRNA/miRNA pathways	Human, Dm, Ce
CCR4-CAF1-NOT complex	Deadenylation	Human, Dm, Sc
CPEB	Translational control	Human
FAST	S/T phosphoprotein activator of Fas	Human
TTP	ARE-mediated mRNA degradation	Human
Staufen	mRNA localization	Human, mice, Dm
Rbp1	Mitochondrial RNA degradation	Sc
Rbp4	RNA pol II subunit	Sc
Sbp1	Suppressor of deccaping	Sc
Germin 5	Part of small nuclear RNPs	Human
Dcs2	Stress-induced regulator	Sc
APOBEC3G, APOBEC3F	Antiviral activity	Human
